# Chronic Laryngitis

**Published:** 1910-07-09

**Authors:** 


### Laryngology and Rhinology.

### CHRONIC LARYNGITIS.

This common and troublesome complaint is often the result of the persistence of an acute catarrh or of frequent recurrent attacks of acute inflammation; but it also not infrequently begins and continues as a chronic affection without the manifestation of any acute symptoms. The most important factors in its causation are nasal obstruction and inflammatory affections of the nose, such as the various forms of rhinitis and suppuration in any of the accessory nasal sinuses. An ill-ventilated, and especially a dusty, atmosphere has a strongly predisposing effect. In addition, certain general constitutional disturbances are important, and must not be overlooked if treatment is to be successful; the chief of these is dyspepsia, especially combined with the abuse of alcohol or tobacco and oral sepsis. The latter is usually associated with carious or badly kept teeth, tartar, or pyorrhœa alveolaris, and mouth-breathing is also a definite cause of septic mouth. A red line can generally be seen on the gum in the neighbourhood of a defective tooth running backwards to the fauces, and a band of injection is often to be observed on the latter parallel to the anterior pillar.

Rheumatism and gout are frequent constitutional causes of chronic laryngitis. The affection is most troublesome in those who use the voice professionally, and here incorrect production is a more fruitful source of ill than over-use. Without going into a dissertation on voice-production, it may be said that the two commonest errors are that of forcing the voice in the larynx without making sufficient use of the nasal resonance combined with stiffness of the muscles of the jaw and throat, and incorrect phrasing without taking breath, whereby phonation is continued with the chest emptied of air. The latter is more frequent among speakers than singers, but it is not uncommonly found that a singer, who produces perfectly while singing, makes use of faulty methods in conversation. The writer has had under his care a singer and teacher of singing, who had to do a large amount of talking and who suffered from chronic catarrh; the production while singing was very good, but the treatment made no progress until attention was drawn to her defective use of the speaking voice. Nasal obstruction exerts a bad influence on the larynx in many ways; by permitting dry, cold, unfiltered air to reach the larynx, by maintaining a chronic infective catarrh of the nares which is liable to spread down the throat, by encouraging oral sepsis, and, above all, by preventing the due employment of the nasal resonators.

The principal symptom is impairment of the voice, which is hoarse and quickly tired, or, it may be, completely aphonic. In the slightest degrees, which only come for treatment in singers, there may be merely some degree of uncertainty in the execution of florid passages or about the middle registers. The hoarseness is usually worse in the evening after use, but in the forms associated with nasal disease and mouth-breathing it is often at its worst on rising and improves after breakfast. There may be a feeling of swelling, dryness, or tickling, and there is often a cough; if much expectoration be present, it comes from the trachea and bronchi, or from the nares and naso-pharynx.

The objective appearances are very variable, and in the slighter grades there may be nothing abnormal to see except that the cords have lost their healthy pearly lustre and look glazed and yellowish, or they may be of any shade of colour from pink to a deep red. The vocal cords are often slightly thickened and rounded, instead of sharp, at their margins, the vocal processes may be unduly prominent and injected, or else may appear white in contrast to the reddened cord. The mucous membrane over the posterior commissure is frequently relaxed, and, having lost its elasticity, becomes wrinkled on adduction of the cords. The thickening here and on the vocal processes may be observed in all degrees up to well-marked pachydermia, which, indeed, is a further development of chronic laryngitis. In the same way the little thickenings, which occur on the centre of the cord in voice-users, and are known as singers' nodules, are due to chronic inflammation, and are really part of a chronic laryngitis, a point to be remembered when their treatment is under consideration. The larynx may be dry and glazed, or there may be hypersecretion; a little globule of mucus may be seen to dance on the cord during phonation, or strings of sticky mucus may stretch across the glottis. The tension and movements of the cords may be impaired, or there may be adductor paresis, partly the result of inflammatory infiltration of the mucosa and muscles and partly functional paresis induced by the increased effort required for phonation.

*Laryngitis sicca* is the name given to a form, usually associated with rhinitis sicca or atrophica and dry pharyngitis: small brown crusts adhere to the cords and interarytenoid region. In *atrophic laryngitis* the crusts are large and often fœtid, and green, brown, or black in colour, like those of atrophic rhinitis, of which disease it is an extension; the crusts may extend down the trachea and cause severe, and even fatal, dyspnœa.

The treatment of chronic laryngitis must be directed to the cause; local measures are of minor importance. In professional voice-users the first thing is to pay attention to production, though it must be confessed that this is a somewhat difficult subject on which to offer advice to an experienced singer. The faults in production already mentioned should be looked for, and an attempt made to correct them. The exercise known as "pmawing" is of great value. The nose must be carefully examined and any obstruction treated; good vocal production is impossible with blocked nasal passages. Vocal rest is of the utmost importance until the larynx returns to normal, and without this good results are impossible. The digestion must receive attention, and in the plethoric a morning saline, with an occasional blue pill, is of great value. Strychnine is indicated when the muscles act badly, and iron in anæmic cases. In the dry forms, small doses of ammonium carbonate, ipecacuanha, and squills are useful, and a stimulating laryngeal spray may be given, preferably an oily solution, containing oleum pini sylvestris or some similar drug. Local applications are rarely advisable, never unless there are hypertrophic changes; chloride of zinc may be used, 15-30 grs. to the ounce, or silver nitrate, beginning with 5 grs. to the ounce, and increasing to 20 grs. or more; the latter is painful, but is said to be efficacious if continued daily, or every other day, for a considerable time.

